# Hypoxia in Vascular Networks: A Complex System Approach to Unravel the Diabetic Paradox

**DOI:** 10.1371/journal.pone.0113165

**Published:** 2014-11-19

**Authors:** Yérali Gandica, Tobias Schwarz, Orlando Oliveira, Rui D. M. Travasso

**Affiliations:** 1 Center for Computational Physics, Department of Physics, University of Coimbra, Coimbra, Portugal; 2 Heinz-Brandt-Schule, Berlin, Germany; 3 Center of Ophthalmology and Vision Sciences(COCV), Institute for Biomedical Imaging and Life Sciences (IBILI), Faculty of Medicine, University of Coimbra, Coimbra, Portugal; University of Arizona, United States of America

## Abstract

In this work we model the extent of hypoxia in the diabetic retina as a function of the area affected by vessel disruption. We find two regimes that differ on the ratio between the area of disrupted vasculature and the area of tissue in hypoxia. In the first regime the hypoxia is *localized* in the vicinity of the vascular disruption, while in the second regime there is a *generalized* hypoxia in the affected tissue. The transition between these two regimes occurs when the tissue area affected by individual sites of vessel damage is on the order of the square of the characteristic irrigation length in the tissue (the maximum distance that an irrigated point in the tissue is from an existing vessel). We observe that very high levels of hypoxia are correlated with the rupture of larger vessels in the retina, and with smaller radii of individual sites of vessel damage. Based on this property of vascular networks, we propose a novel mechanism for the transition between the nonproliferative and the proliferative stages in diabetic retinopathy.

## Introduction

Diabetes occurs when the pancreas does not secrete enough insulin or the body is unable to process it properly [1]. High levels (and/or wide fluctuations) of glucose concentration in the blood can lead to a number of complications, including an increased risk of cardio-vascular disease, kidney disease (nephropathy), neural damage (neuropathy), and retinal disease (diabetic retinopathy, DR) [2]. The number of deaths attributed to diabetes is about 9% of the global total of deaths [3].

Diabetic retinas suffer extensive blood vessel disruption (e.g. the formation of defects such as micro-aneurisms, and dramatic vessel proliferation increase, leading to the formation of small blood lakes and to loss of vessel function) [4] which stems from the inflammatory environment that is associated with high levels of methilglyoxal [5], and lead to the production of several proteins that have a vascular destabilization action. An example is the angiopoietin-2 (Ang-2) [6] that binds the receptors Tie-2 at the membrane of the vessels' endothelial cells, thus preventing the mediation of angiopoietin-1 in the maturation of the capillaries. Therefore, in the absence of pro-angiogenic factors, high levels of Ang-2 induce an increase of vessel permeability, with the consequence of a deficient tissue irrigation [7] (note, however, that the loosening of the Tie-2 junctions between endothelial cells is also an important step of the angiogenic process, and therefore, Ang-2 is able to promote angiogenesis depending on the presence of pro-angiogenic factors). It has been shown experimentally in mice that retinal vascular network damage is able to drive neo-vascularization characteristic of the later stages of DR [8], [9].

A large body of work has been done to understand the pathogenesis of diabetic microvasculopathy [10], [11]. Most of these efforts have been centered in the mechanisms related to specific signaling pathways [12], [13]. Nevertheless, we argue in the present work that the spatial distribution of areas with deficient irrigation, i.e. in hypoxia, also play an important role in the determining the progression of the disease. In these regions, the lack of oxygen leads to higher levels of angiogenic factors that promote the remodeling of the vasculature as well as the growth of neo-vessels, both hallmarks of DR [14]. In consequence, the study of how the distribution of hypoxic regions depends on vascular damage is essential for the understanding of DR's progression. In this work we will focus on the changes in irrigation after events of vessel blockage, inspired by the effect of factors such as Ang-2, that are present in high levels in a DR setting, and that have the potential to drive extensive vessel damage rendering the smaller vessels non-functional. This study is closely related with the analysis of resilience in spatial complex networks, where is investigated the effect of a particular amount of damage in the nodes and/or links on the integrity of the complex network.

The resilience of complex networks to damage has attracted a great deal of attention in the past years [15]–[23]. Most of the studies on this topic link the random or selected removal of nodes to the alteration in the structural properties of networks and have important applications. For example, the study of the percolation properties of a network of social relations upon node removal can be applied directly to viral epidemics. In this case, random node removal gives information about the amount of agents to immunize in order to prevent the propagation of the epidemics [24]. Also, the consequences of the breakdown of power lines resultant from a natural hazard, for example, is a problem that can be formulated in terms of the failure of interconnected networks, see e.g. [25], [26].

Networks are studied by defining the nodes of the system and the connectivity between them by means of edges or bonds. Other specific characteristics can be added according to the system that is modeled, such as bond weights, directionality, etc [15]–[17]. Typically, when structural and dynamic characteristics of a network are determined solely by their connectivity, it can be considered to be embedded in an abstract “network space” [27]. However, when networks have the role of delivering energy, water or nutrients, the physical space constraints are important. In these networks, resilience after the rupture of bonds at a particular location is connected to the lack of goods, energy, water or nutrients in spatial regions, and not to the number of nodes and bonds affected per se [28]. Not surprisingly, the applications of the study of the resilience in spatial networks are vast, however most previous studies in resilience of complex networks have not considered spatial constraints [27].

Vascular networks are a particular type of spatial network constituted by two different topologies of connections that fulfill different functions [29]. Larger vessels, arterioles and venules form *specific* fractal tree-like networks [30]–[33] that are responsible for blood transport [34]. In these structures, alterations in the branching pattern [35], branching lengths [36], [37], fractal dimension [32], [38] among others, are often an indication of a pathology. On the other hand, nutrient and oxygen diffusion is carried out mainly by capillaries which are laid down in space approximately in a lattice-like structure [29], [39], [40]. Vascular networks are dynamic, since they can adapt to the nutrient requirements of the tissue they irrigate [41]. Therefore, spatial constraints, such as the nutrient and protein diffusion, affect not only the tissue cells survival, but also the structure of the vascular network itself.

Earlier studies on the hypoxia in damaged vascular networks showed a localized decrease in irrigation after specific targeting of blood vessels. Blinder et al. [30] studying robustness of distribution of blood in rodent neocortex to occlusion experiments found that removing 15% of the connections in the backbone isolates 5% of the cortex from perfusion. More recently, the same authors investigated experimentally the volume of micro-infarctions in the rat brain, resultant of the occlusion of specific arterioles [42], demonstrating that even small volumes of low irrigation have important cognitive consequences.

Our aim is to relate the different stages of DR with the distribution of tissue irrigation decrease after a total loss of vascular function in localised regions in the retina. This article is organized as follows. In the next section the retina vessel network is described, together with the details of calculation of the tissue irrigation. In the Simulation and Results section we investigate how the affected irrigation changes with the damaged vasculature. In the Discussion section the results are analyzed and its physio-pathological implications to the development of DR are discussed. Finally, in the last section we summarize and conclude.

## Materials and Methods

**Figure 1:**
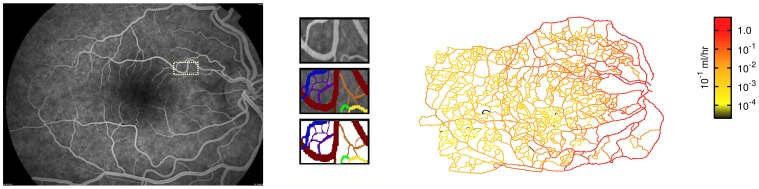
Characterization of the vascular network. Left panel: The original angiography of the retina that was digitalized. Identified with a small rectangle is the area that is amplified in the central panel. Center panel: Zoom of detail in angiography: original detail (top); manual identification of vessels with different colors indicating joined vessels of similar caliber (center); geometry that is digitalized to obtain the reconstructed network (bottom). Right panel: The blood flow in the reconstructed network. The color gradient indicates the flow intensity.

The aim of this work is to propose a mechanism relating the evolution of the proliferative stage of DR with the patterns of hypoxia in the retinal tissue after damage of its vascular network. Therefore, we start with a vascular network that mimics the retinal vasculature. The top layer of the vasculature at the retina is almost two dimensional [43], so that its approximate structure can be obtained from the digitalization of an angiography. In this work we used an angiography of a human retina (with 1684×1192 lattice units, equivalent to 9.4×6.6 mm; each lattice unit corresponds to 5.6 µm), obtained from a patient in the early stages of diabetic retinopathy (left panel in Fig. 1). Patients at Coimbra's University Hospitals routinely perform angiographies to assess the progress of different diseases (normally DR or age related macular degeneracy). This results in an image where the deficiencies of the retinal vascular network can be clearly visualized. The hospital stores several of these images obtained from anonymous patients as example of the different stages of relevant pathologies. We chose one image from that set corresponding to a very early stage of DR, where the pathology-driven vasculature alteration was the least perceptible. No human tissues or cells were used in this study.

A closed vascular network is obtained extending manually the thinner arterial vessels to intercept the thinner veins (see Fig. 1 center). Taking into account the thickness and contrast in the original angiography, we are able to manually identify the sites where the vessels overlap without establishing bifurcations. Even missing the thinnest capillaries, this network provides the basic structure of in-vivo retinal networks. The measured number of bifurcations between the main vessel (artery or vein) and the furthermost capillary is 35. This number is of the same order of magnitude as the number of successive vessel bifurcations observed in higher organisms [44].

The blood flow in the network (right panel in Fig. 1) is approximated by assuming Poiseuille flow in all vessels, i.e., that they have cylindrical symmetry and that the blood viscosity is high enough to neglect turbulence and inertial effects [44]. For the human body, this is a good approximation, except for the larger arteries and veins [44]. At every bifurcation in the vessel network we apply blood flow conservation, i.e. the sum of incoming flows to a node matches the sum of the outgoing flows. In all calculations we also disregard the alterations in blood viscosity due to the Fahreus-Lindqvist effect and the viscoelasticity of the blood (in the Text S1 we demonstrate that the inclusion of the Fahreus-Lindqvist effect does not affect in a significant way our results).

Our analysis is based on a steady state study, and therefore we do not take into account the pressure and radii variation at the larger vessels due to the heart beat. Also, given that most of the flow in the retinal vessels is laminar, solving the full Navier-Stokes equation can only change the pressure at the vessels for a small amount, leaving essentially unchanged the calculated hypoxia regions after vessel suppression.

Very high levels of Ang-2 increase the vessel permeability, and, in the limit, render them incapable of irrigating the tissue. In this work we take this limit, and consider the total loss of function of individual thin vessels in the regions affected by vessel damage. We use a simplified method to estimate the effects of vascular disruptions on tissue oxygen levels. In reality, the oxygen level declines nonlinearly with the distance along each vessel, depending on the flow rate, the oxygen consumption rate, and on the nonlinear oxygen binding characteristics of hemoglobin. For simplicity, we here use intravascular hydrostatic pressure as an index of position along each flow pathway, and assume a linear dependence of the oxygen concentration on pressure, setting the levels of oxygen at the retinal artery equal to 1 and the concentration at the veins equal to 0. Since the model used is linear in the levels of oxygen in the tissue, the profile of hypoxia observed is independent of the values of the oxygen levels at the arteries and veins, if the critical oxygen concentration for hypoxia in the tissue is altered accordingly. In fact, we expect that the observed hypoxic regions after vessel suppression will depend mainly on the diffusion of oxygen in the tissue (and in particular on the characteristic irrigation length, i.e. the maximum distance that an irrigated point in the tissue is from a vessel, function of the oxygen diffusion length, the oxygen consumption rate and the critical oxygen concentration for hypoxia), and not on the details of how the oxygen levels at the vessels are calculated (see below and Text S1).

The transport of oxygen to the tissues occurs via a diffusive process. We consider that the oxygen consumption rate in the tissue is constant and, therefore, the time-independent concentration of oxygen 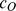 can be obtained from the Poisson equation 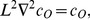
(1)


where 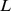 is the tissue oxygen diffusion length. In this equation, we linearize the Michaelis-Menten kinetics normally used for the consumption of oxygen by the tissue [45], [46], since the regions of hypoxia that are analyzed in this work are determined by the characteristic irrigation length (see below and Text S1), i.e. by the levels of oxygen far from the immediate vicinity of the capillaries, which is where the non-linear behavior of the Michaelis-Menten is relevant.

Equation (1) is solved in a large rectangular domain that includes the totality of vessel network. We use the successive over-relaxation method setting the concentration of oxygen to zero at the borders of the domain, and having as boundary condition the concentration of oxygen at the vessels that are able to irrigate. The capillary endothelium is highly specialized to permit the exchange of oxygen between the blood and the tissue. In contrast, main arteries and veins have the function of transporting the blood to and from the capillaries [34] and have thick walls that often require their proper irrigation to be oxygenated. For this reason, we consider that only the thinner vessels (venules, arterioles and capillaries) with flow larger than zero can irrigate (in the model we assign this function to vessels with diameter smaller than 24 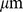 so that most of the vessels in the simulation participate actively in irrigation of the tissue). Therefore, when equation (1) is solved for every point in the tissue, the concentration of oxygen is fixed as boundary condition at the smaller vessels (with diameter smaller than 24 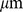) if their flow is larger than zero.

**Figure 2:**
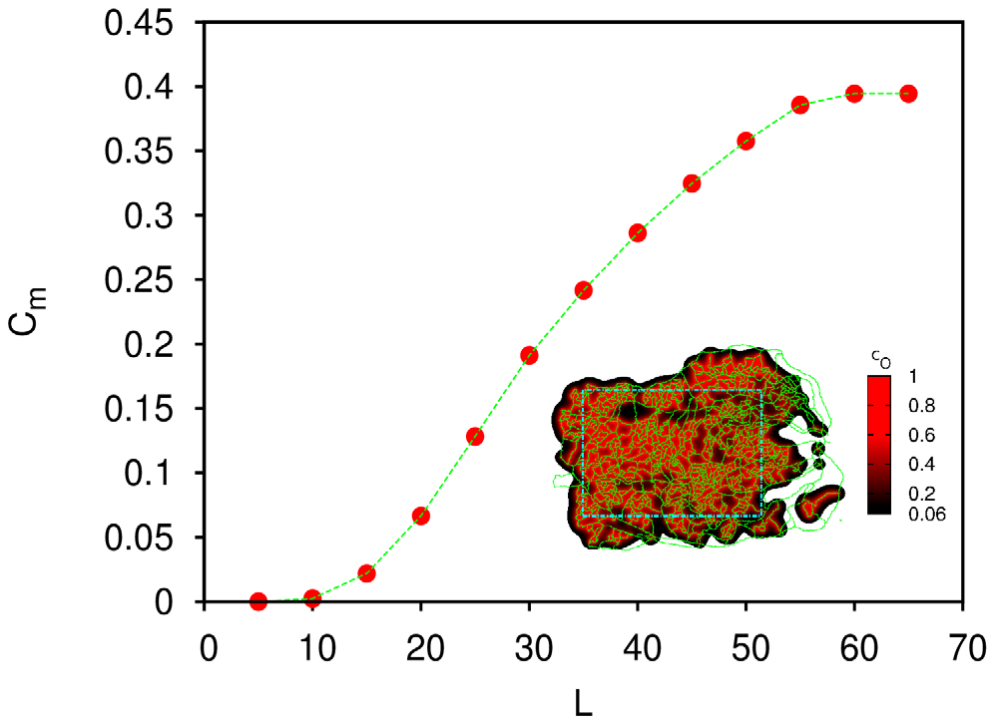
Plot of the 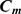 required for the normoxia of all tissue inside the rectangle (inset) as a function of the diffusion length of oxygen, 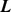. Inset: In this diagram we represent the vascular network analyzed (in green), the 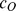 in the retina (in the gradient black-red) for 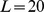 lattice units and 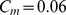, and the boundary of the region of the retina analyzed in this work (in turquoise).

If 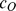 falls below a critical value, 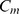, the tissue cells will enter in hypoxia. In Fig. 2 we show the maximum value for 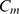 such that all the tissue region highlighted in the inset (inside the turquoise dashed rectangle) is in normoxia as a function of 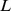 (since a site is in normoxia if 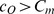, the plot corresponds to the minimum value of 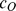 inside the highlighted region as a function of 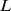). In order to avoid the irregular boundary of the network, we will focus on the rectangular region highlighted in the inset of Fig. 2 in the rest of the work. The lower the value 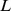, the faster equation (1) is solved numerically. We chose 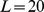 lattice units (112
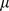
m) and 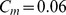 (corresponding to a point on the beginning of the rising part of the curve in Fig. 2) for faster run times and we have found that our results are independent of this particular choice for 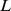 (see also Text S1).

## Results

To simulate the effect of localized vascular loss of function of the thinner vessels, we consider small circular regions of radius 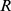 in the vascular network where the vessel damage occurs. Inside these regions, we block all vessels with diameter smaller than a cutoff, 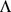. These circular regions are distributed randomly within the highlighted area in the inset of Fig. 2. The area fraction covered by these blocking regions will be deemed 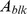. In what follows we will take 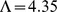 lattice units, i.e. 24 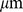, the average of the diameters of the vessels in our network. The results will be compared to those using a larger cutoff 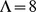 lattice units, i.e. 45 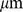. Physiologically, taking larger values of 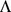 means considering also the suppression of thicker vessels.

Once the vessels are blocked, the blood flow will be altered (i.e. we re-calculate the flow, now in the altered vessel network), the irrigation of the retina will be lower and this may lead to new regions in hypoxia. We measure how the total area in hypoxia changes with 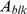, the area where the thin vessels are blocked.

**Figure 3:**
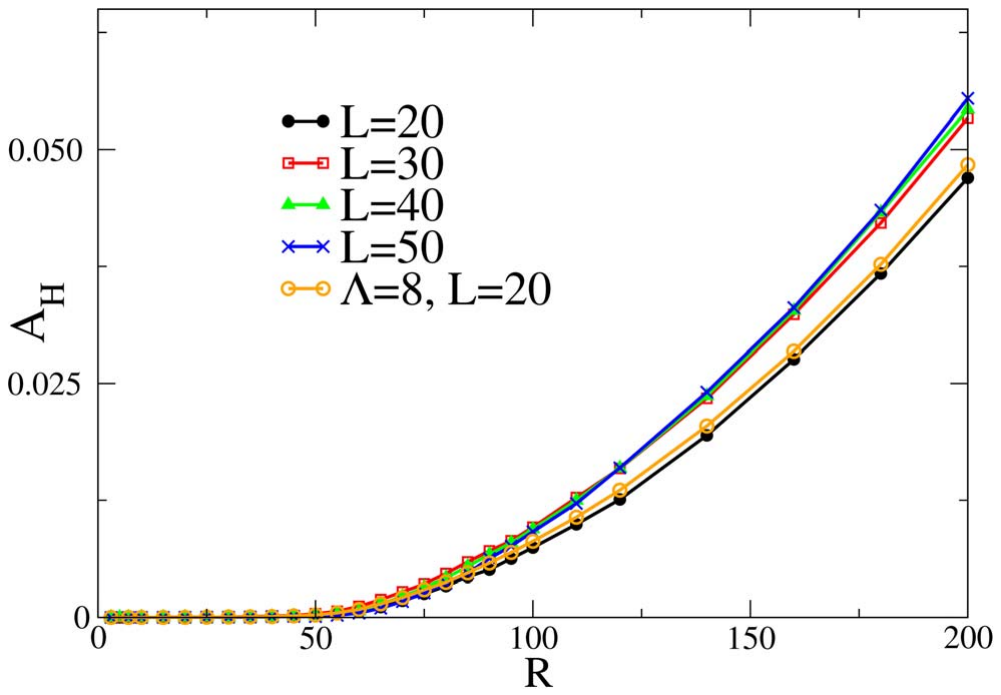
Resulting area in hypoxia for one blocking spot of radius 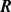. Each curve has a different diffusion length, 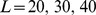 and 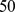 (each point in the graphic represents the average of 500 runs). Also shown are the results for 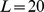 with 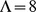. The curves for different 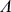 and 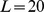 are, essentially, undistinguishable. In all cases the radius of the circle necessary for an average hypoxia region larger than zero is 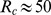.

Fig. 3 depicts the average area in hypoxia, 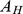, for only one blocking circular area of radius 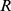. The data in the plot are the average over 500 random realisations of the blocking sites. The picture shows the results for different values of diffusion length 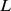 and two different cutoffs 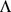. Interestingly, for all these different parameters there is a single critical value of the radius (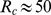) below which only one blocking circle does not induce an appreciable change in the irrigation of the network. It is therefore necessary a minimal size of the blocking target to produce an area in hypoxia. We observe that this characteristic distance in the system does not depend on the value of 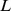 or 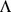, but on the maximum distance that a point is from a capillary vessel in the analyzed network (see Text S1). Hence, 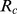 will be designated by the characteristic irrigation length.

**Figure 4:**
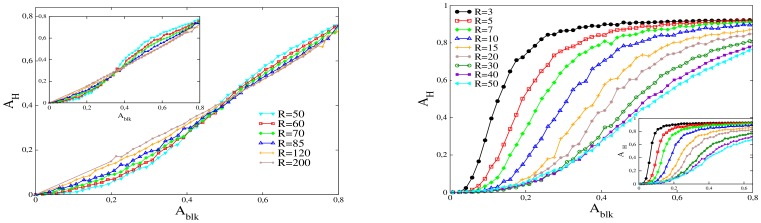
Fraction of area in hypoxia (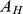) versus fraction of area vessel disruption in the retina (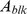). Left panel: Plots for 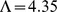 and radii of each blocking circular neighborhood larger than 50 lattice units. The inset corresponds to 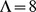, i.e. when vessels with diameter smaller than 8 lattice units are compromised. Right panel: Same as in the left panel, but for radii smaller than 50.

We proceed by increasing the number of damaged circular neighborhoods, which are distributed randomly (they may overlap). We calculate the fraction of area in hypoxia, 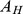, as a function of the area fraction of retina affected by the vessel disrupting proteins, 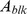, see Fig. 4. Each curve corresponds to vessel damaged regions built with blocking circles with the same radius 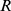. The radii are indicated in lattice units. For equal blocked areas the hypoxia can be considerably different, depending on the radii of the blocking regions.

We observe a monotonic increase of 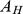 with 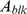 for all the values of 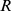. In general, for large blocked areas, a larger 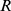 corresponds to a smaller area in hypoxia. However, for small values of 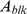 we found a different behavior depending if 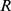 is larger or lower than the characteristic irrigation length, 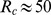. Above this radius (Fig. 4 left), at blocking areas below a critical area 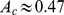, and contrary to what happens for 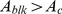, a larger 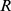 corresponds to a larger area in hypoxia. On the other hand, for 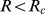 (Fig. 4 right) the area in hypoxia is independent of the radius for very low 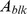, i.e. all the curves collapse into a single curve at low 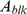 (the section of the curve where this collapse occurs depends on the value of 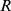, with a larger overlap region for larger values of 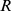). Besides, for 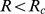, the value of 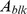 at which the transition to the large area regime occurs is lower than 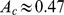 and strongly dependent on the radius, in opposition to the situation where 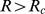. This transition for 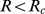 is associated with a very steep increase in 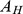. The slope of this increase is larger for smaller values of 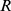.

## Discussion

### Damage and Hypoxia

To understand the dependence of the 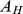 on 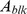 and 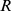, we will start by analyzing how hypoxia regions are formed after vessel blockage in this system.

**Figure 5:**
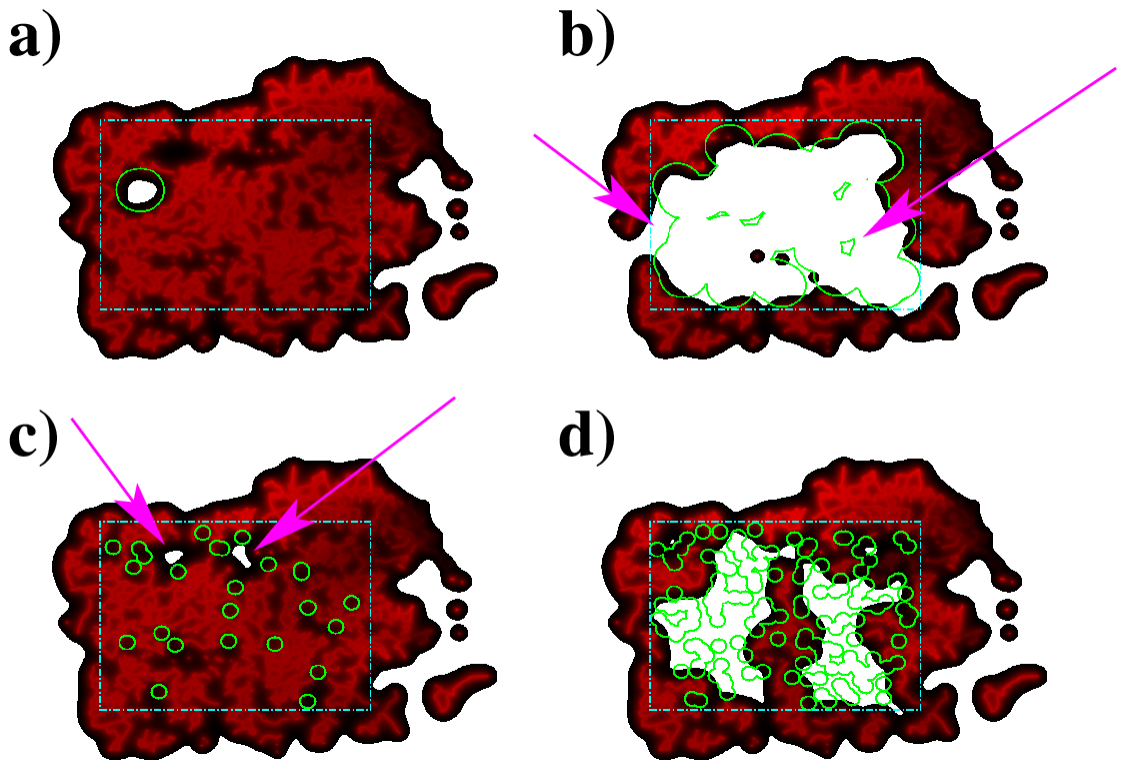
Irrigation, i.e. the density plot of 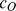, in different scenarios. a) one blocking circle with 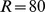, b) 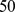 blocking circles with 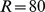 (arrows signal sites with no irrigation or vessel damage), c) 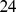 blocking circles with 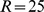 (arrows signal areas in hypoxia resultant from the combined action of several blocking sites) and d) 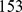 blocking circles with 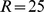. For each plot, the blocked regions are in green, the local irrigation are represented by the color gradient, from black (lower) to red (higher) and the areas in white inside rectangle of interest have 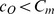, and are in hypoxia.

The blockage induced by a single circular region with radius larger than the characteristic irrigation length produces an area in hypoxia which is smaller than the blocked region (Fig. 5a). Points inside the blocking region that are at a distance larger than the characteristic irrigation length 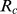 from the circumference of the region, are in hypoxia. Hence, for blocking circles with 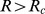, the area that is still irrigated is approximately equal to 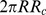, i.e. it grows linearly with 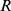 and not with 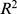. Therefore, for larger blocking circles, the areas inside each circle that are still irrigated are proportionally smaller. Consequently, for 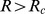, for a small number of these regions and for the same covered area, we observe that higher values of 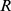 will correspond to a higher hypoxic area (see Fig. 4 left).

As the number of blocked areas gets larger, 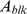 increases until the system reaches a regime in which regions are not irrigated, in spite of not having their vessels blocked. This is the consequence of being surrounded by the circular blocking areas (Fig. 5b)). In this regime, the number of such oxygen deprived regions will be larger if 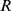 is smaller, since for smaller 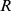 the number of circular blocking areas is higher, for the same 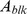. With a higher number of circular regions becomes easier to surround not affected areas, blocking the required vessels to deprive those regions of irrigation. Therefore, if 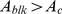, smaller 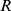 will correspond to larger 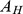, in opposition to what is observed for 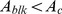.

A completely different scenerio occurs for 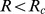, i.e. when we are covering the network with targets smaller than its characteristic irrigation length. In this regime the effect of covering areas loses its local nature, because a single blocking region can not create hypoxia (as seen in Fig. 3). Hypoxia can only be reached with the cooperating effect of many blocking regions. This can be clearly seen in Fig. 5c, where the few hypoxic areas are the result of the particular disposition of various neighboring blocking spots.

As the number of blocking spots increases, there will be a density at which these spots have a high probability for blocking vessels separated by 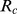. Above this density, that corresponds to a critical area 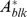, hypoxia will occur everywhere in the tissue. An example of such a situation is in Fig. 5d. We expect a sharp transition between 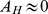 and almost total hypoxia at this particular density of blocking sites. For 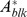 the area in hypoxia will be dramatically larger than 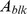. This effect can be clearly seen in the steep slope of the curves in right panel of Fig. 4.

It is possible to obtain the dependence of the critical area 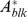 on 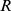 for very small 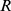, disregarding overlap between the circles. Consider a blocking region of radius 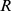 much smaller than 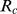. To have hypoxia, circles have to block vessels that are located at least at a distance 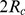. One of these circles blocks a given vessel, if its center is closer to the vessel than a distance 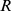. The associated probability for cutting the vessel is then of the order 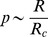. On the other hand, in 2D the number of blocking regions in a square of side 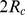 will be 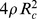, where 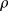 is the number of blocking circles per unit area. In order to have hypoxia everywhere, the critical 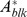 should be such that is able to block the vessels, i.e. the probability of blocking a vessel is on the order of unity. Hence, the minimum density of points required, 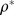 obeys 
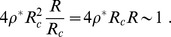



We approximate 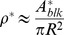, obtaining finally that 
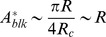
, i.e. the blocking area at the steepest slope in the right panel of Fig. 4 has a linear dependence with 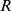.

For 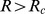 to have a large hypoxic region, the distance between the blocked centers should be on the order of the blocking circles radius, i.e. 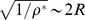. Then, 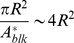 and for 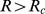, 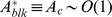 is independent of 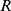.

**Figure 6:**
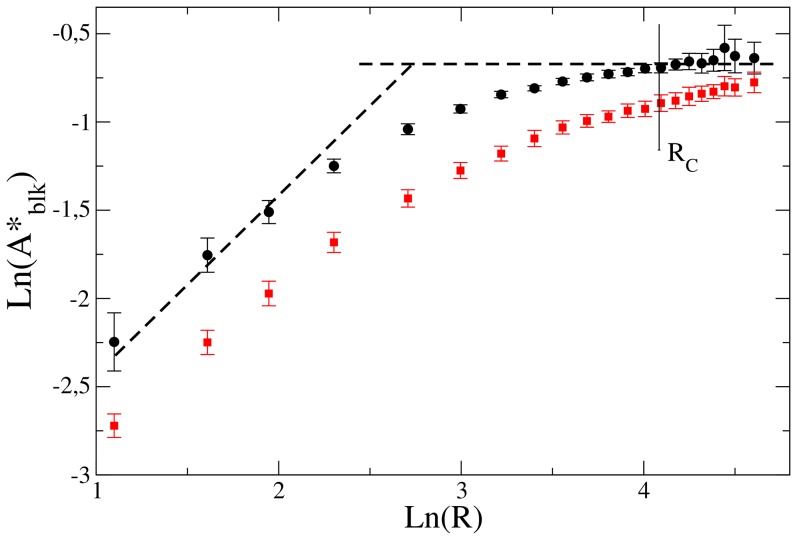
In black circles, the logarithm of inflexion points, from curves showed in Fig. 4, versus the logarithm of 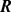. We compare to two straight dashed lines with slope 1 and 0. We observe that the transition points follow 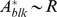 for 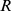 much smaller than 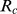 and 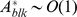 for 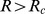. In red squares the same for 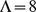 lattice units.

In Fig. 6 we show the log-log graph of the inflexion points for the curves plotted in Fig. 4. The inflexion points are obtained through a fit of the curves to an hyperbolic tangent function superimposed on a linear function. The error bars correspond to the statistical error of the non-linear fit. We associate the inflexion points to the transition values for 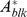. We can see that the dependence of 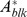 is linear for 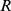 much smaller than 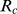, becoming independent of 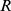 for 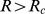, in good agreement with the predictions discussed above.

For 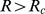 the size of the blocking circles are larger than any characteristic scale in the underlining network, and the irrigation of the tissue will be blocked only when the blocking circles percolate the system. We also verify the non-dependence of 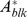 on 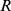 which is characteristic of the circular blocking neighborhoods percolation (see Text S1). Therefore in this regime the transition does not depend on the underlining network.

### Consequences for DR progression

We observed that the complex effects of covering areas with targets smaller than the lattice parameter produces hypoxic regions that can grow easily larger than the affected area for 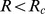. This regime can have important consequences in the progression of the disease.

It is a known fact that DR can be divided in two main stages with different phenotypes. The earliest stage of DR is nonproliferative diabetic retinopathy (NPDR). Though this stage can be itself divided in different sub-stages [1], [2], it is characterized by the presence of micro-aneurisms (swellings in capillaries and arterioles), a variable degree of vessel blockage and the consequent deprivation of irrigation in some regions of the retina. The most advanced stage of DR, the proliferative diabetic retinopathy, PDR, is characterized by neovascularization, the growth of abnormal (fragile and leaky) new blood vessels, which may lead to blindness through hemorrhage and scarring [47]. This pathological retinal angiogenesis generates randomly orientated and physiologically deficient vessels that do not conform to neuronal histology [14].

The creation of this abnormal and dense structures is rooted in the following paradox (often referred in the literature as the diabetic paradox [48], [49]): if the prevalent action in DR is the regression, disruption and blockage of blood vessels, then how does the hallmark of the most advanced stage of the disease is a very high vascular density? Does the fact that the vascular disruption sites in DR are extremely small and localized [50], [51] play an important role in the progression of the disease?

According to our study, the blockage of vessels, akin to what happens in DR, leads to a deficient irrigation of the tissue, and to an increase of hypoxia in the retina. However, a tissue in hypoxia leads to an increase in the production of the HIF-1 (Hypoxia Inducible Factor) transcription factor, which has angiogenic factors, such as VEGF, as targets. The consequence is the triggering of neovascularization in the tissue [52]–[54]. We expect that when this neovascularization occurs repeatedly in regions where vessel regression and blockage occurred, the increase in VEGF levels will occur locally, balancing the effects of the antiangiogenic factors that led to the vessel disruption. The result will be a vasculature with the same physiological density and caliber but with possible local malformations [55]. In these situations we expect to find a NPDR phenotype.

However, when the disruptions of the vasculature lead to hypoxia in regions with little or no vessel regression, the neovascularization resultant from the high VEGF levels in these regions will not counterbalance the original disruption. Therefore, the process of angiogenesis would lead to a denser vasculature. In these situations we expect to find a PDR phenotype.

As we mentioned, in the right panel of Fig. 4 we observe a regime where the area in hypoxia is larger than the affected area. In this regime, associated with low values of 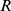 and large 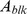, we clearly have areas that will be producing very high levels of VEGF, since they are in hypoxia, and still have functional vessels. This setting suggests a high vessel density as consequence, and we can associate this effect with a PDR phenotype.

Therefore, we predict that the PDR phenotype occurs when 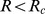 as the density of affected points increases, i.e. as the disease progresses. This will happen in sections where a fraction of the retina larger than 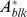 is affected. Besides, the existence of very small and localized sites of vascular disruption in DR in vivo is in agreement with the hypothesis of 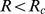 being required for PDR to develop [50], [51].

However, we found that there is a possibility to decrease 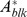 and to obtain the PDR regime at lower affected areas. In the insets of Fig. 4 we show 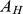 as a function of 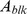, but now we change the cutoff of the system to 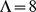 lattice units. In this case in each blocking region all vessels with radius smaller than 8 units will be compromised, i.e. thicker vessels may also be affected. In spite that, in this case the minimum radius 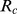 of a blocking circles that can induce hypoxia is still the same as it is shown in Fig. 3. Accordingly, we still observe in the inset of Fig. 4 left and in Fig. 6 the approximately constant value for 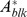 for 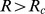. Of course that, as 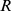 grows, the value of 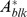 should reach 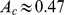, the value associated with the percolation of the blocking circles in this system. However for 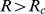 the increase of 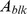 is extremely slow (Fig. 6).

For all values of 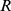 we observe that the values of 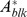 are much lower when the thick vessels are also affected. Even for the largest 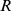 in the left panel of Fig. 4 the value 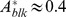 is lower than the case where only the capillaries are affected. This is consequence of the specific structure of the retina network. The largest vessels are few in number but have the important role of distributing the blood through all the network. Cutting these vessels lead to a larger loss in irrigation than cutting only the small capillaries. The consequence is a lower value for the critical 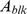.

In what concerns the progression of DR, we anticipate that the PDR phenotype can dominate in two scenarios: either the affected area increases, or the type of vessels affected is altered and larger vessels are also compromised. In both situations the phenotype is most severe when the radius of each focus of damage is smaller, for the same affected area.

## Conclusion

We analyzed the effect of vessel damage in the retina irrigation, consequent of the high levels of vessel destabilizing proteins, such as Ang-2, in a diabetic environment. In this study we modelled blood flow and the oxygen concentration in the tissue. Although more exhaustive methods exist to describe oxygen pressure dynamics [45], [56]–[59] and the flow in vascular networks [60], the present model captures the main ingredients of the processes involved. However, when more subtle vessel disruption events are considered, (e.g. increases in the permeability of the capillaries that do not compromise the vessel viability, partial blockage of vessels), a more complete description of oxygen transport needs to be implemented, including how the oxygen levels decline nonlinearly with distance along each vessel as a function of the flow rate, and how it is consumed in the tissue according to a Michaelis-Menten kinetics [45], [58], [59]. The study of oxygen levels in these situations complement the work presented, and will be the subject of a future study.

Having access to the basic topology of the vascular network of a human retina, we found two regimes delimited by a critical size of blocking spots radius. This critical radius corresponds to the characteristic irrigation length in the network. Furthermore, these results suggest that the prevalent phenotype of the pathology can be a direct consequence of the distribution of disrupted and hypoxic regions in the tissue. We associate the situation where the hypoxia occurs prevalently in the region of vessel disruption with the non-proliferative diabetic retinopathy phenotype. On the other hand, if the extensive regions of hypoxia occur outside the region of vessel disruption we expect a proliferative diabetic retinopathy phenotype.

Our results suggest that disrupting the vessels in the retina with targets of radius lower than the characteristic irrigation length in the tissue (typical in DR [50], [51]), may lead to large hypoxia levels with relatively low values of vessel destabilizing areas. These areas will be even lower if the vessel destabilizing environment is able to rupture thicker vessels. These results convey a new way of thinking about the diabetic paradox [48], [49]: allowing the possibility that localized vessel disruption points lead to the formation of large regions in hypoxia, which have the ability to drive neovascularization [52], [53], excessive angiogenesis is able to occur in pathologies that are driven by regression and blockage of blood vessels.

These conclusions on the mechanisms for the early development of the two phenotypes of DR may be of high importance in the definition of prevention strategies for the development of DR is risk patients.

## Supporting Information

Text S1 **In this text we discuss several details of our model.** We focus on the relation between the characteristic irrigation length and the geometry of the network, on the relevance of modeling the Fahreus-Lindqvist effect, and on the relation between the percolation transition and the transition from the non proliferative phenotype to the proliferative phenotype.
(PDF)
