# Supplementary material for: Hypoxia in Vascular Networks: A Complex System Approach to Unravel the Diabetic Paradox
Source: PLoS One. 2014 Nov 19;9(11):e113165. doi: 10.1371/journal.pone.0113165 (PMC4237512; doi:10.1371/journal.pone.0113165)
Supplement: Text S1 — In this text we discuss several details of our model. We focus on the relation between the characteristic irrigation length and the geometry of the network, on the relevance of modeling the Fahreus-Lindqvist effect, and on the relation between the percolation transition and the transition from the non proliferative phenotype to the proliferative phenotype. (PDF) [file pone.0113165.s001.pdf]

Supporting Information

Characteristic Irrigation Length

The considered vessel network has a characteristic irrigation length that depends on its geometry. The capillaries and small vessels define a lattice that divides space into domains of various sizes. The histogram of the domain sizes, measured as the square root of the domain areas, can be seen in the next figure for the vascular network used in our study.

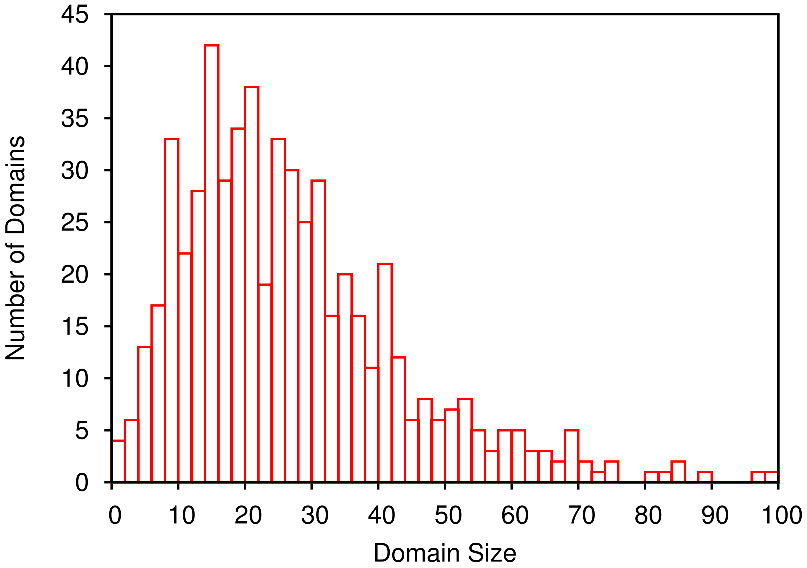

It follows that the characteristic domain in the network has the linear size of 20 lattice units. This value is the lattice parameter of the system of the vascular network but does not present a constraint to the irrigation of the tissues. The cut-off, C_m, for the concentration of oxygen is set to guarantee normoxia, and is the lowest value that permits the irrigation of all points in the tissue. Therefore the irrigation is related to the maximum distance between a point in the tissue to a capillary or a vessel.

For our network, the larger domains are 100 lattice units wide and, therefore, the furthest that a point is from a capillary is about 50 lattice points. It follows that, independently of the value of L, the furthest distance from a capillary that is irrigated, i.e. the characteristic irrigation length, is about half the size of the largest domain in the lattice, 50 units.

Irrigation and the Fahreus-Lindqvist effect

The apparent viscosity of the blood is lower for the smaller vessels. In these vessels, the solid component of the blood flows closer to their center, leaving the liquid component as the main responsible for the friction exerted at the vessel wall, resulting in a lower apparent viscosity. This is called the Fahreus-Lindqvist effect. In spite of being a crude approximation to disregard the Fahreus-Lindqvist effect when calculating blood flow and pressure in a vessel network, we demonstrate that this effect does not play an important role for the calculation of the hypoxia areas after complete vessel suppressions.

In the figure below, we repeat the same exercise as in Fig. 4 of the main article but considering the Fahreus-Lindqvist effect. We consider that the viscosity depends on the blood vessel diameter according to what is observed experimentally. We use the values for the viscosity as a function of the vessel diameter observed for thin tubes and reported in Fig. 1A of the article [1]. The graphics we obtain are very similar to Fig. 4 of the main article.

By simulating the Fahreus-Lindqvist effect, we alter considerably the pressure at the vessels. Nevertheless we saw no appreciable difference in the hypoxia area after blockage. We reason that when a vessel is blocked it affects strongly the oxygen flow in its neighborhood since it stops irrigating. The area affected depends strongly on the characteristic irrigation length and not on the details of how the blood and/or the oxygen pressure at the vessels is determined. In other words, when vessels are blocked in a vascular network that was able to irrigate a region in the tissue, the

direct loss of oxygen delivery from the blocked vessels is much more determinant for hypoxia than the changes in the pressure of oxygen in the vessel network that result from the blockage. Therefore, equation (1) from the main text, together with the critical concentration of oxygen for irrigation, C_m, capture the relevant physics for the study of hypoxia resultant from total suppression of vessels.

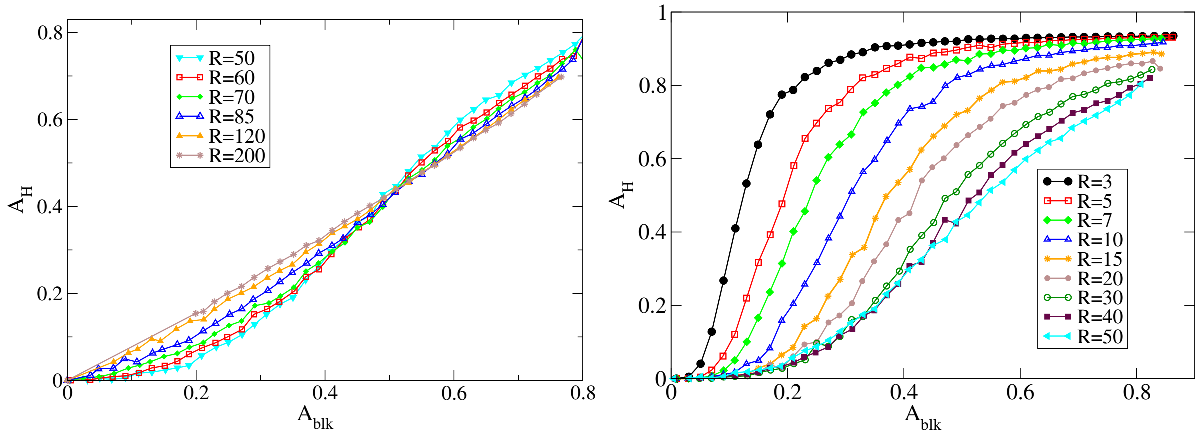

Percolation in a circle covered region

For comparison with the results described in the main text, let us look at percolation of circular regions in a domain of the same size as the area investigated (1684×1192 lattice units) but without taking into account the vascular network.

Starting from this rectangle, we randomly distributed a number of circles with a specific radius ($R = 10, 30, 50, 70$ in this case). We then measured the area occupied by this configuration of circles and observed if there was percolation in the system. Recall that percolation occurs when opposite sides of the rectangle are connected by the covered region. We repeated this exercise for different numbers of circles, taking note of the covered area and of the existence or not of percolation, for each case. To obtain a graphic with the probability for percolation as a function of the covered area, we divided the total area of the rectangle in bins with width 0.02 of the total area. From all the runs completed, we counted for each bin the fraction of those runs where percolation was observed. In the next figure we plot this probability for percolation as a function of the covered area.

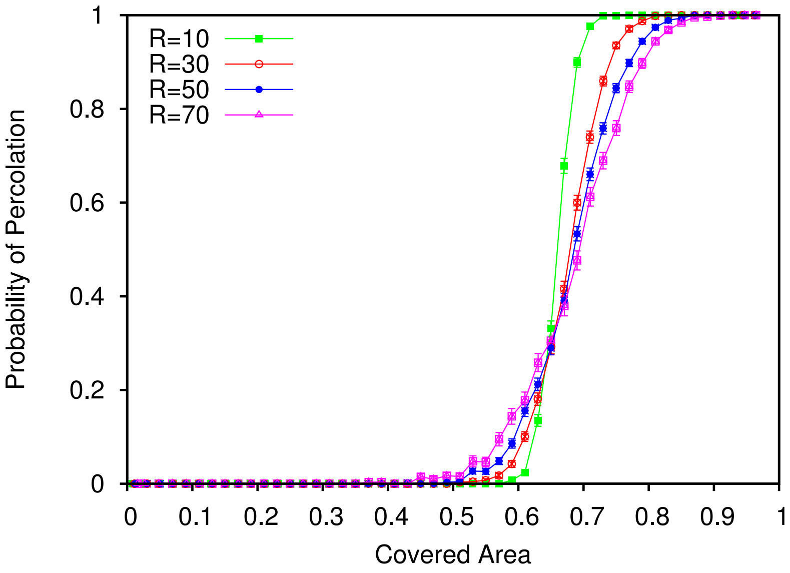

The figure shows a transition for all radii. The smaller the radius, the steeper is the curve, with all transitions occuring at approximately the same covered area. The curves have the same qualitative behavior as that reported in the main text for the hypoxia area associated with large radii. Note that if one ignores the vascular network, the regime where the critical area varies with R observed previously for $R < R_c$ does not occur anymore.

In the system with the vascular network, when there is percolation of the blocked regions there will be large drop in the irrigation of the tissue (notice that this drop in irrigation will not be total since we are blocking vessels just inside the rectangle of interest and blood flow can enter in this rectangle through various points in its boundary). For large radii, the characteristic irrigation length is smaller than R, and the vessel network has little importance in the determination of hypoxia. In fact, the area in hypoxia will be directly related to this percolation of the blocked areas, and one can expect a behavior for A_H vs A_{blk} which mimics a percolation type of transition. The transition will not be as abrupt as a pure percolation transition because for $R > R_c$ the hypoxia grows linearly with the blocked area for small A_{blk}. However, at the percolation transition the area in hypoxia grows steeply, since large parts of the domain become isolated. The fact that the curves in Fig. 4 (left) associated to different R cross all at the same point can therefore be viewed as a a signal of the percolation of the hypoxia produced by the blocking regions.

In Fig. 4 (left) the transition occurs at a lower fraction of the occupied area, A_c, compared to the transition of the pure percolation problem. In the retina network, two vessel blocking areas do not need to overlap to block the passage of blood (see Fig. 5b). Due to the underlining vascular network, the neighboring circles only need to cut neighboring vessels to cut the blood supply in the space between them. Therefore, in the system with the vascular network, the percolation of hypoxia will occur at a lower value of blocked area.

[1] Sugihara-Seki, M., & Fu, B. M. (2005). Blood flow and permeability in microvessels. Fluid dynamics research, 37(1), 82-132.
